# Identifying Knowledge Barriers for Smart Home Blood Pressure Monitoring Device Usage Among Minority Older Adults

**DOI:** 10.1093/geroni/igaf122.3342

**Published:** 2025-12-31

**Authors:** Yanjun Dong, Jany Sun, Jimmie Boliboun, Katherine Koo, Valeria Vazquez-Trejo, Michael Cui, Victoria RIzzo, Jeannine Rowe

**Affiliations:** SUNY at Albany, Albany, New York, United States; Rush Medical College, Chicago, Illinois, United States; Rush Medical College, Chicago, Illinois, United States; Rush University Medical Center, Chicago, Illinois, United States; Rush Medical College, Chicago, Illinois, United States; Rush Medical College, Chicago, Illinois, United States; University at Albany, SUNY, Albany, New York, United States; University of Wisconsin-Whitewater, Whitewater, Wisconsin, United States

## Abstract

Background Hypertension remains a critical health concern among minority older adults, with disparities in management and outcomes. Smart Home Blood Pressure Monitoring Devices (SHDs) offer a promising tool for improving hypertension management, yet adoption remains limited. This study examines knowledge barriers affecting SHD use and their impact on health outcomes and healthcare engagement. Method This qualitative study employed semi-structured interviews with 20 minority older adults (aged 60+), primarily African American and Latinx, diagnosed with hypertension. Participants were recruited from a health equity study at an academic medical center. Data were analyzed using thematic analysis through ATLAS.ti, guided by Andersen’s Expanded Behavioral Model of Health Services Use. Results Knowledge was identified as a key predisposing factor influencing SHD adoption. 60% of participants reported no prior awareness of SHDs before being prescribed the device, with healthcare providers serving as the primary information source. Knowledge gaps, including device functionality and data security uncertainty, contributed to inconsistent use. Among those unfamiliar with SHDs, 42% reported negative experiences, and 33% reported psychological barriers. Despite initial challenges, 90% reported regular use after receiving guidance, with self-monitoring and blood pressure management improvements. Discussion Healthcare providers can bridge knowledge gaps through proactive and continuous patient education. Culturally tailored educational interventions and digital literacy programs can improve self-efficacy and support sustained device usage. Incorporating SHD education into chronic disease management programs and leveraging community networks may enhance adoption. Future research should explore the long-term effects of these interventions on SHD usage and health outcomes across diverse older adults.

